# *YangZheng XiaoJi* exerts anti-tumour growth effects by antagonising the effects of HGF and its receptor, cMET, in human lung cancer cells

**DOI:** 10.1186/s12967-015-0639-1

**Published:** 2015-08-27

**Authors:** Wen G. Jiang, Lin Ye, Fiona Ruge, Sioned Owen, Tracey Martin, Ping-Hui Sun, Andrew J. Sanders, Jane Lane, Lucy Satherley, Hoi P. Weeks, Yong Gao, Cong Wei, Yiling Wu, Malcolm D. Mason

**Affiliations:** Cardiff University-Peking University Cancer Institute, Cardiff University School of Medicine, Henry Wellcome Building, Heath Park, Cardiff, CF14 4XN UK; Yiling Medical Research Institute, No. 238 TianShan DaJie, Shijianzhuang, HeBei Province China

**Keywords:** Lung cancer, Tumour model, *YangZheng XiaoJi*, Hepatocyte growth factor, cMET, HGF, Cellular migration

## Abstract

**Background:**

Hepatocyte growth factor (HGF) is a cytokine that has a profound effect on cancer cells by stimulating migration and invasion and acting as an angiogenic factor. In lung cancer, the factor also plays a pivotal role and is linked to a poor outcome in patients. In particular, HGF is known to work in combination with EGF on lung cancer cells. In the present study, we investigated the effect of a traditional Chinese medicine reported in cancer therapies, namely *YangZheng XiaoJi* (*YZXJ*) on lung cancer and on HGF mediated migration and invasion of lung cancer cells.

**Methods:**

Human lung cancer cells, SKMES1 and A549 were used in the study. An extract from the medicine was used. Cell migration was investigated using the EVOS and by ECIS. Cell–matrix adhesion and in vitro invasion were assessed. In vivo growth of lung cancer was tested using an in vivo xenograft tumour model and activation of the HGF receptor in lung tumours by an immunofluorescence method.

**Results:**

Both lung cancer cells increased their migration in response to HGF and responded to *YZXJ* by reducing their speed of migration. *YZXJ* markedly reduced the migration and in vitro invasiveness induced by HGF. It worked synergistically with PHA665752 and SU11274, HGF receptor inhibitors on the lung cancer cells both on HGF receptor activation and on cell functions. A combination of HGF and EGF resulted in a greater increase in cell migration, which was similarly inhibited by *YZXJ,* and in combination with the HGF receptor and EGF receptor inhibitors. In vivo*, YZXJ* reduced the rate of tumour growth and potentiated the effects of PHA665752 on tumour growth. It was further revealed that *YZXJ* significantly reduced the degree of phosphorylation of the HGF receptor in lung tumours.

**Conclusion:**

*YZXJ* has a significant role in reducing the migration, invasion and in vivo tumour growth of lung cancer and acts to inhibit the migratory and invasive effects induced by HGF and indeed by HGF/EGF. This effect is likely attributed to the inhibition of the HGF receptor activation. These results indicate that *YZXJ* has a therapeutic role in lung cancer and that combined strategy with methods to block HGF and EGF should be considered.

**Electronic supplementary material:**

The online version of this article (doi:10.1186/s12967-015-0639-1) contains supplementary material, which is available to authorized users.

## Background

Lung cancer is the leading cancer worldwide and results in more death than any other cancer types. In the UK, the incidence of lung cancer in males has steadily declined, but in contrast, the rate in females has steadily increased [1–3]. Worldwide, although the incidence rate has been declining in developed countries, since the later part of the last century, the incidence continues to rise in countries in which smoking has peaked including China, Korea, and several countries in Africa and will continue to do so for the next few decades [2, 4]. Treatment options for lung cancer remain limited, which is reflected in the poor survival, namely 5 year survival remains below 10 % and 10 year survival close to 5 %. Surgery, chemotherapy and radiotherapy remain the main treatment choice, although new targeted therapies are now available, namely anti-EGFR, etc. [3, 5, 6].

Recently, there have been early clinical reports that a Chinese medicinal formula, known as *YangZheng XiaoJi (YZXJ)*, has a beneficial effect in patients with lung cancer [7, 8]. In a number of small trials, *YangZheng XiaoJi,* in combination with chemotherapy has been shown to increase the survival rate and at the same time, reduced the side effects. A similar beneficial effect has been reported in patients with primary hepatocellular carcinoma [9]. Although it was initially proposed that the beneficial effects may be due to the improved immune function, such as the increase in NK cell functions, there have been recent reports to show that *YangZheng XiaoJi* was able to directly inhibit angiogenesis and migration of cancer cells, including osteosarcoma cells, an effect attributable to the inhibition on the activation of focal adhesion kinase [10, 11].

Hepatocyte growth factor (HGF) is a cytokine that has strong effects on normal cells and cancer cells [12, 13]. In normal physiology, the cytokine is involved in tissue regeneration and organ repair, for example liver and lung regeneration. In cancer, however, the cytokine has been shown to have a profound effect on the migration, invasion and growth of cancer cells and has acted as a powerful angiogenic and lymphangiogenic factor [14, 15]. In the majority of solid tumour types, HGF and its receptor, cMET, have been found to be over-expressed in cancer cells and tumour tissues. It has been shown to be linked to disease progression, metastasis and long term clinical outcome of the patients [15–17].

In non-small cell lung cancer (NSCLC), HGF receptor protein over-expression has been frequently demonstrated [18, 19] and is shown to be associated with a poor clinical outcome of the patients. It has been shown that cMET protein expression is increased in NSCLC lung tumours with ALK gene rearrangement [20], and that gene amplification is uncommon in lung cancer. The amplified cMET protein expression may be the result of transcription factor ETS2 which was frequently down regulated in lung cancer [21].

In lung cancer, HGF has also been shown to interfere with EGF tyrosine kinase activation, which in turn results in induced resistance to EGFR inhibitor therapies [22]. Thus, combined use of MET tyrosine kinase inhibitor (TKI) and EGF TKI has been suggested to be a valid novel combination to overcome TGF TKI acquired resistance in lung cancer [23]. This was indeed shown in an in vitro study in which the cMET small inhibitor E7050 has the ability to circumvent resistance to the reversible, irreversible, and mutant-selective EGFR-TKIs induced by exogenous and/or endogenous HGF in EGFR mutant lung cancer cell lines, by blocking the Met/Gab1/PI3 K/Akt pathway in vitro [24]. It is interesting to note that HGF-positive serum is a predictive factor for patients negative response to gefitinib therapy with advanced NSCLC who harbour wild-type EGFR [25, 26]. Serum HGF levels have been shown to be linked to disease progression and overall survival, and interestingly even more so when EGFR status was considered [27].

cMET protein over-expression was seen in more than half of small cell lung cancer (SCLC) and patients with cMET phosphorylation in the SCLC tumours have a markedly poor overall survival (132 vs 287 days for those with cMET phosphorylated tumours and non-phosphorylated tumours respectively, p < 0.001) [28]. Circulating HGF (cHGF) was found to be a regular feature for patients with lung cancer and has been suggested to be a useful biomarker in choosing a HGF/cMET based targeted therapy [29, 30]. Lung cancer may attract neutrophils to release their HGF storage and contribute to the local microenvironment for lung cancer progression [31].

Previous reports have demonstrated a profound effect of *YangZheng XiaoJi* on the migration of cancer cells and indeed on endothelial cells, suggesting that the medicine is an important regulator for cell motility and potential target for therapy [32, 33]. HGF is one of the most powerful motogens (motility stimulating cytokines) which, together with the HGF receptor, cMET, are aberrantly expressed in lung cancer. In the present study, we sought to investigate the effects of *YangZheng XiaoJi* on the migration, invasion and cell adhesion of lung cancer cells, in particular in the context of stimulation by HGF. In this context, the degree of the activation/phosphorylation of the HGF receptor was also investigated in order to decipher if and how *YangZheng XiaoJi* might affect the HGF/cMET complex. The study also examined the effect of *YangZheng XiaoJi* on the growth of lung tumours in vivo. Here, we report that *YangZheng XiaoJi* has an inhibitory effect on the functions and growth of lung cancer cells, in vitro and in vivo. *YangZheng XiaoJi* also exhibited an effect on HGF- and HGF/EGF-induced cellular migration on lung cancer cells and impacted on the phosphorylation of the HGF receptor, cMET.

## Methods

### Cells and materials

Human lung cancer cell lines, SKMES1 and A549 were obtained from ATCC/LGC standard (Teddington, Middlesex, England, UK). Recombinant human hepatocyte growth factor was a kind gift from Professor Toshikazu Nakamura (Osaka University, Osaka, Japan). Recombinant human EGF was from Sigma Aldrich (Poole, Dorset, England, UK). Small inhibitors to HGF receptor, SU11274 (IC_50_ for cMET 20 nM) and PHA665752 (IC_50_ for cMET 9 nM), and small inhibitor to the EGFR kinase, AG490 (IC_50_ for EGFR 2 µM) were obtained from Tocris Chemicals (Bristol, England, UK). Antibodies to human HGF, human cMET (SC-10), and phospho-cMET (sc-34088) were obtained from Santa Cruz Biotechnologies Inc., (Santa Cruz, CA, USA). Horse radish peroxidase (HRP) conjugated fluorescently tagged secondary antibodies were obtained from SigmaAldrich (Poole, Dorset, England, UK).

The herbal medicinal formula, *YangZheng XiaoJi* was obtained from Yiling Pharmaceuticals (Shijiazhuang, HeBei, China). The formula contained the following 16 ingredients: *Panax ginseng C.A. Mey.*, *Astragalus memebranaceus* (*Fisch.*) *Bge.var. mongholicus* (*Bge.*) *Hsiao, Ligustrum lucidum Ait.*, *Curcuma phaeocaulis Val.*, *Ganodema lucidum*, *Gynostemma pentaphylla* (*Thunb*) *Mak*, *Atractylodes macrocephala Koidz*, *Scutellaria barbata D.Don, Oldenlandia diffusa* (*willd.*) *Roxb., Poria cocos, Duchesnea indica Focke, Solanum lyratum Thunb., Artemisia scoparia* (*Bge.*) *Ki, Cynanchum paniculatum Kitag*, *Eupolyphaga sinensis Walker*, and *Gallus domesticus Brisson*. The chemical fingerprints of different batches were found to be highly consistent. Shown in Additional file 1 are fingerprints from 10 batches of *YangZheng XiaoJi* over a 2 year period (Additional file 1).

*YangZheng XiaoJi* extract, referred to as DME25, was prepared from *YangZheng XiaoJi* as previously reported using a DMSO based method [8, 10, 34]. Briefly, *YangZheng XiaoJi* formulated powder was added to pure DMSO solution (Sigma Aldrich). This was placed on a rotating wheel (Labinco BV, Wolf Laboratory, York, England, UK) for 12 h in a cold room at 4 °C at 100 rpm [8, 10]. The preparation was centrifuged at 15,000×*g,* for 20 min at 4 °C. The supernatant was carefully removed and filtered using a disc filtration unit (pore size 0.20 μm, Sartorius Stedim, Sartorius, Epson, Surrey, England, UK). The extract was diluted in a balanced salt solution (BSS) and standardised by quantifying the optical density of the preparation using a spectrophotometer at 450 nm wavelength (Biotek, Wolf Laboratory). A master preparation which gave 0.25 OD at 450 nm was stored as the master stock and so named as DME25 for the subsequent experiments. DME25 at dilutions below 1:40 did not show an effect on the growth of lung cancer cells. In the present study, we used the dilution range between 1:100 to 1:2000.

### Electric cell-substrate impedance sensing (ECIS) based cell adhesion and cell migration assays

This was modified from a method previously described [35, 36]. Briefly, 96-well W96E1 microarrays were used on the ECIS Ztheta instrument (Applied Biophysics Ltd, Troy, New Jersey, USA). Lung cancer cells were added to the wells of the array, followed by immediate tracking of cell adhesion over a range of frequencies, namely from 1000 to 64,000 Hz using automated modules. The adhesion was analysed using the mathematical modelling methods as previous described [37]. For cellular migration, confluent lung cancer monolayers in the arrays were electrically wounded (2000 mA for 20 s each), after which the migration of the cells was immediately tracked, again over a range of frequencies. All the experiments were conducted in triplicate.

### EVOS cellular migration assay (wounding assay)

Cellular migration assays were conducted on an EVOS system (EVOS^tm^ fl Digital Inverted Fluorescence Microscope, Fisher Scientific, Paisley, Scotland, UK). The human-interface free system integrated automated digital time lapse recording, temperature and air control, automated sampling of same sample point over a specified time and over large number of replicates (up to 96 wells).

Cells were seeded into a 96-well plate (Nunc, Fisher Scientific, Paisley, Scotland, UK) at 10,000 per well and allowed to reach full confluence overnight in the incubator. The monolayer was then scratched using a fine tipped plastic pipette to create a wound of approximately 200 µm wide. The media together with the floating cells were removed and replaced with media containing either*YangZheng XiaoJi* extract alone (1:1000), or in combination with HGF (50 ng/ml) or HGF receptor inhibitor (PHA665752 10 nM, SU11274 20 nM, based on their IC_50_ value). Each treatment was done in quadruplicate wells. The plate was immediately placed in the chamber of the EVOS unit (which was programmed to supply 5 % CO_2_ and maintained at 37 °C constant temperature). The EVOS was programmed to memorise the three dimensional position of the wounding position in each well and then automatically tracked the image of each well, every 15 min for up to 9 h. Sequential images were analysed using Image J software. Four images were randomly chosen, one from a replicate at the beginning of the experiment and at the end of each hour. Due to the nature of the wound front (leading front of the wounded cell monolayer), 10 points were chosen randomly in each image and the distance between the opposing fronts at each point was then measured. Together, distance at 40 points for each treatment was quantified. The net distance that the cell migrated at each point was calculated as: distance migrated = (T0 − Tn)/2, where T0 is the distance between two leading wound edges at the beginning of the experiment, and Tn is the distance between the wound leading edge of the same wounding point at a given time and at a given point (38). The distance is shown as the mean number of pixels that cells had migrated from the 4 replicates. The migration is shown as a time course, and the comparison between the experimental settings was based on a fix time point.

### In vitro invasion assay

Invasion assays were performed based on a previously published method [38]. Transwell invasion inserts with pore size at 8.0 µm were first coated with 50 µg Matrigel and allowed to air dry. The gel was then carefully rehydrated. To each insert, 30,000 cancer cells were added together with the test agents or their combinations. After 72 h, Matrigel and non-invading cells were removed using a cotton swab. Invaded cells on the lower surface of the inserts were fixed with 4 % formaldehyde and then stained with 1 % crystal violet. The number of invaded cells were counted under a microscope.

### SDS PAGE and western blot

Lung cancer cells at sub-confluence were first subjected to serum hunger for 4 h in a serum free culture medium. The test agents including HGF (50 ng/ml), DME25 (1:1000), PHA665752 (10 nM), or their combinations were respectively added to the cells for a period of 45 min. Sodium orthovanadate (100 µM) with hydrogen peroxide (0.1 %) was used as a positive control. Cells were collected using a cell scrapper. After centrifugation (1000 rpm for 5 min), media were removed from the cell pellets. Proteins from the cell pellets were extracted using a lysis buffer that contained 1.5 % Triton X100 as detergent, and protease inhibitors. Protein concentrations were standardised. Equal amount of proteins were loaded onto 8 % SDS PAGE. After separation, proteins were blotted onto nitrocellulose membrane, which were subsequently probed by specific antibodies to total MET, phospho-MET (p-MET) and GAPDH. The protein blocking solution contained 1 % horse serum. After probing with horse-radish peroxidase conjugated antibodies, the protein signal was detected using chemiluminescence methods (EZ ECL, Cellseco Ltd., Salisbury, Wiltshire, England, UK) and images were taken on a luminescent/fluorescent imager (Syngene, G: Box, Cambridge, England). The protein band signal was quantified using Image-J software. Shown here as pMET/cMET ratio.

### In vivo tumour model

Athymic female nude mice, CD-1 of 4–6 weeks old were purchased from Charles River Laboratories (Margate, Kent, England, UK). Due to the sensitive response to DME25 and the HGF receptor inhibitors, we chose A549 cell line for *the* in vivo tumour model. Tumour cells, which were prepared at 5 × 10^6^/ml in a solution containing 3 mg/ml Matrigel, was injected subcutaneously to give 0.5 million cells per injection [39, 40]. After one week when tumours became visible, mice were randomly divided into groups (n = 6 in each group). The size of tumours were measured using digital callipers. Treatments began one week after tumour cell inoculation and included the following: Control group (receiving control buffer), *YangZheng XiaoJi* extract via intraperitoneal injection (IP) or by gavage (oral), cMET inhibitor (PHA665752), a combination of *YangZheng XiaoJi* extract and cMET inhibitor. Treatments were given daily. The final concentration of *YangZheng XiaoJi* extract was 1 µl/g body weight and PHA665752 6.4 ng/gram body weight. Tumour size was measured weekly for 4 weeks. Mice were weighed and monitored daily. The procedure was reviewed and approved by the Cardiff University Joint Biological Ethics Committee and conducted under the UK Home Office Project License (PPL 30/2591). At the conclusion of the experiments, mice were terminated by euthanasia. Tumours were dissected and immediately stored at −80 °C for subsequent analysis. Tumour volumes were calculated by the following formula: tumour volume (mm^3^) = length × width × 0.54 [41].

### Immunofluorescence staining

Frozen tumours were sectioned using a cryostat (Leica CM1900) at 7 µm thickness. After fixation using acetone/methanol fixation buffer, the slides were rehydrated and blocked with a Tris buffer (25 mM, pH 8.4) that contained 10 % horse serum for 1 h. Primary antibodies (including anti-cMET, anti-pMET), diluted in the blocking buffer was added to the respective slides which were kept in a humidified box for 1 h, and then thoroughly washed. FITC- or TRITC tagged secondary antibodies were then added to the respective slides together with DAPI for nuclear counter stain. After 1 h, the slides were thoroughly washed and mounted using FluroSave™ (Calbiochem, Nottingham, England, UK). The slides were examined on an Olympus microscope and photographed using a Hamamatsu digital camera. The staining intensity was determined using Image-J. Briefly, the images were first converted to greyscale andthe staining intensity at the cell–cell junction areas where cMET is located, were calculated from Image-J, minus the reading from a background control. Eight images were calculated from each staining.

### Statistical analysis

Statistical analysis was carried out using Sigma Plot (version 11, Systat Software Inc., San Jose, CA, USA). ANOVA test was used on normalised data and ranked ANOVA was used on non-normalised data.

## Results

### YangZheng XiaoJi had a profound influence on cell–matrix adhesion, cell migration and in vitro invasion

**Fig. 1:**
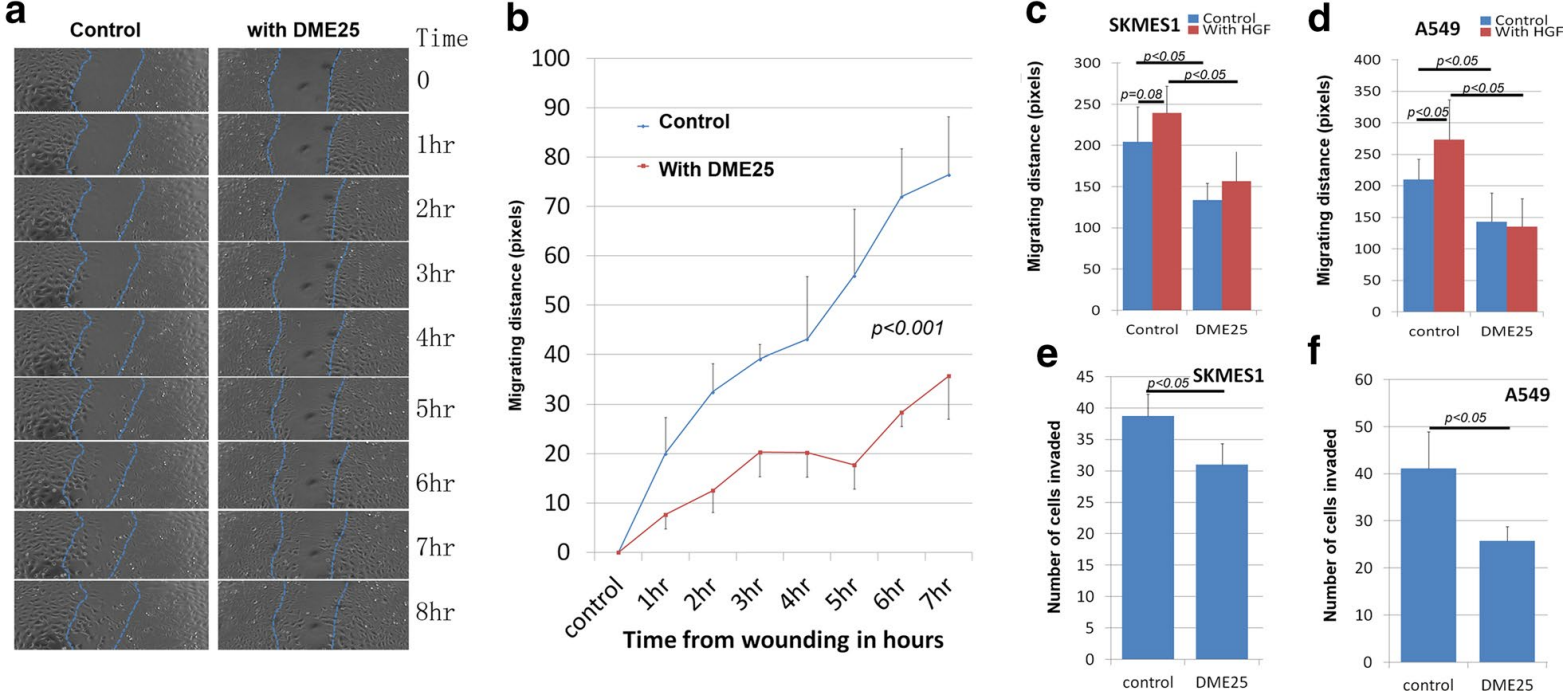
Effects of *YangZheng XiaoJi* on cell migration, adhesion and invasion. **a** Cell images from A549 wounding model. *Left* control group in which cell monolayer was wounding and monitored continuously for 8 h. *Right* DME25 treated group, similarly wounded and monitored. DME25 dilution used was 1:1000. **b** Cellular migration of A549 as tracked by EVOS imaging system and quantified migration speed (in pixels). *YangZheng XiaoJi* had a significant inhibitory effect on the migration (p < 0.001). **c**, **d** *YangZheng XiaoJi* HGF-induced cell migration (shown are at 6 h after wounding). HGF (shown at 50 ng/ml) had a significant stimulatory effects on cell migration of both A549 (**d**) (p < 0.05 vs control) and to some degree SKMES1 cells (**c**) (p = 0.08). *YangZheng XiaoJi* inhibited HGF-induced cell migration (p < 0.05 vs control, p < 0.05 vs with HGF only). **e**, **f** Effects of *YangZheng XiaoJi* on cell invasion. *YangZheng XiaoJi* inhibited in vitro invasion of both cells (p < 0.05 vs control)

The effect of *YangZheng XiaoJi* was firstly tested on cellular migration. Using automatic cell tracking with the EVOS system, it was shown that *YangZheng XiaoJi* significantly reduced the migration speed of lung cancer cells (Fig. 1a, b). As expected, HGF increased the speed of migration of both cancer cells. It is interesting to observe that inclusion of *YangZheng XiaoJi* significantly reduced the pace of migration (Fig. 1c, d). Likewise, in vitro invasiveness was also tested. As shown in Fig. 1e, f, *YangZheng XiaoJi* also significantly reduced invasiveness of both cells.

**Fig. 2:**
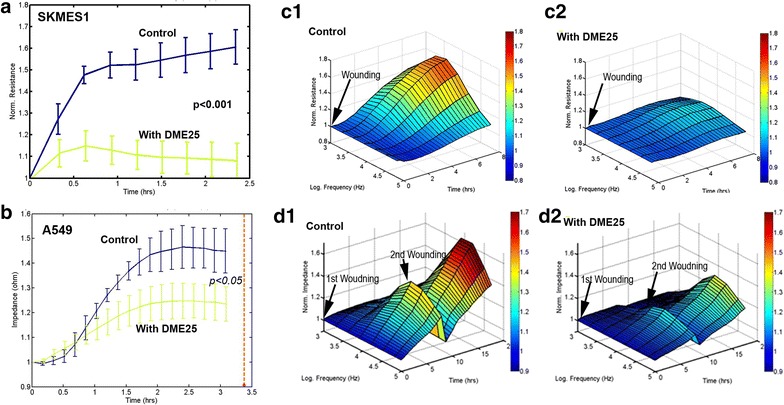
Electric cell-substrate impedance sensing (ECIS) based evaluation of cell adhesion (**a**, **b**) and cellular migration (**c**, **d**). **a**, **b** Evaluation of cell adhesion by ECIS. The ECIS array was first treated with cysteine for 20 min followed by washing. Medium or tested agents were then pipetted to the respective wells, immediately followed by adding cancer cells to each well. The ECIS unit then immediately began tracking the adhesion of the cells. Shown are traces of the adhesion of the respective cells. Cell adhesion of SKMES1 and A549 to matrix was markedly inhibited by *YangZheng XiaoJi* (shown is the extract used at 1:1000). *Error bars* shown are standard deviation (n = 4). *YangZheng XiaoJi* significantly inhibited the adhesion of both cells (p < 0.05, control vs DME25 treatment). **c** Effects of *YangZheng XiaoJi* on the migration of SKMES1 cells analysed by ECIS. SKMES1 cells, after reaching confluence, were electrically wounded (indicated by *arrow*). After replacing the medium with fresh medium contained test agent, the pace of migration was immediately traced. Shown are the cell response in a 3-D model: resistance in y-axis (ohms), over a 7-h period (z-axis) and across 7 frequencies (1000, 2000, 4000, 8000, 16,000, 32,000 and 64,000 Hz) (x-axis). Observations were made with multiple frequencies in order to track if cell behaviour can be viewed differently over a broad spectrum of frequencies. The cellular migration of SKMES1 after electric wounding was suppressed by *YangZheng XiaoJi* (**c2**), compared with control (**c1**). **d** Effects of *YangZheng XiaoJi* on the migration of A549 cells analysed by ECIS. Test condition was the same as SKMES1 in (**c**), except that 7 h after the first round of wounding, a second wounding assay was applied, in order to evaluate the reproducibility of the same cell in the same model (indicated by the 2nd *arrow* in **d1**, **d2**). Similarly, the migration of A549 (**d**) was also inhibited by *YangZheng XiaoJi*. The A549 cells were repeatedly wounded and a similar inhibition was seen (**d**). **c**, **d** *y-axis*: resistance (ohms); *x-axis*: frequencies in Hz; *z-axis*: time in hours

In the study, we also employed another automated method in tracking cell adhesion and migration, namely an ECIS based method. As shown in Fig. 2a, b, *YangZheng XiaoJi* had some marked inhibitory effects on the adhesion of both cells. An electric wounding based method was applied to confluent lung cancer cells followed by tracking the migration of both cells. As shown in Fig. 2c, the migration of SKMES1 cells was markedly suppressed by *YangZheng XiaoJi.* A549 cells exhibited a similar response to *YangZheng XiaoJi,* in that repeated wounding demonstrated a similar response (Fig. 2d).

### YangZheng XiaoJi had a synergistic effect with cMET kinase inhibitors on HGF induced effects on lung cancer cells

**Fig. 3:**
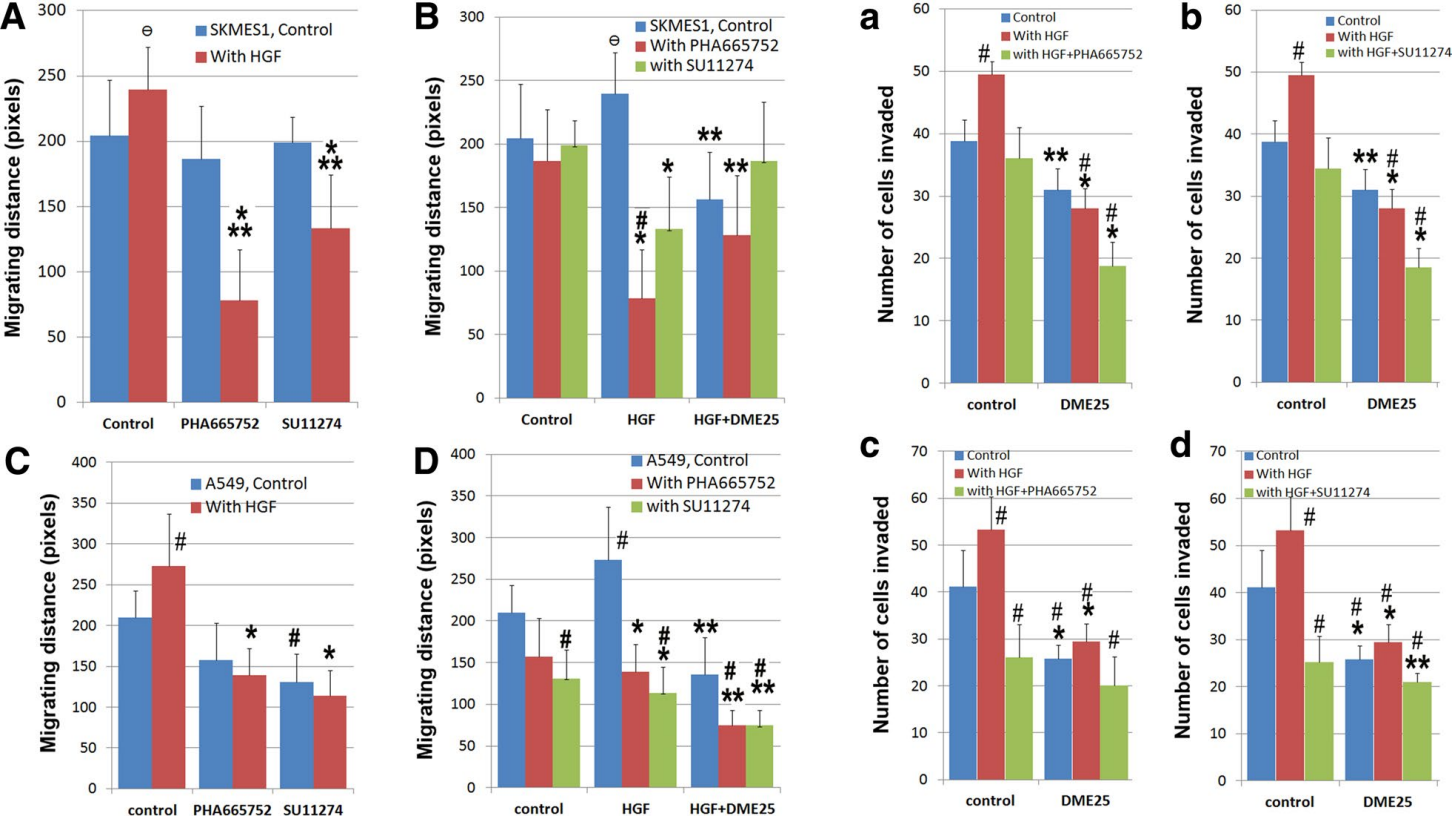
The effect of *YangZheng XiaoJi* and cMET kinase inhibitors, PHA665752 and SU11274 on HGF-induced cell migration (**A**, **B**, **C**, **D**) and invasion (**a**, **b**, **c**, **d**), on both SKMES1 (*top panel*) and A549 (*bottom panel*) cells. *Migration assay* **A** both PHA665752 (10 nM) and SU11274 (20 nM) had a significant effect on HGF-induced migration of SKMES1 cells. HGF concentration shown here is 50 ng/ml. *p < 0.01 cells with HGF/PHA665752 combination vs cells with control medium only; **p < 0.05 cells with HGF/SU11274 combination *vs* cells with HGF only. ^Ɵ^p = 0.059 cells with HGF alone vs cells with control medium. **B** The effect of the combination between DME25 and HGF inhibitors on SKMES1 cells. *p < 0.05 cells with HGF/SU11274 (or HGF/PHA665752) combination vs cells with HGF only; **p < 0.05 cells with HGF/SU11274/DME25 (or HGF/PHA665752/DME25) combination vs cells with HGF only; ^#^p < 0.05 cells with HGF/PHA665752 combination vs cells with control medium only; ^Ɵ^p = 0.059 cells with HGF alone vs cells with control medium. **C** effect of HGF and HGF receptor inhibitors on the migration of A549 cells. *p < 0.01 cells with HGF/SU11274 (or HGF/PHA665752) combination *vs* cells with HGF only; ^#^p < 0.05 respective cells *vs* cells with control medium only. **D** effect of HGF, and the combination of DME25 and HGF receptor inhibitors on the migration of A549 cells. *p < 0.01 cells with HGF/SU11274 (or HGF/PHA665752) combination vs cells with HGF only; **p < 0.05 cells with HGF/SU11274/DME25 (or HGF/PHA665752/DME25, or HGF/DME25) combination vs cells with HGF only; ^#^p < 0.05 respective cells *vs* cells with control medium only. *Invasion assay* **a**, **b** DME25 significantly potentiated the effect of HGF receptor inhibitors, PHA665752 (**a**) and SU121274 (**b**), on HGF-induced invasion of SKMES1 cells. The effects were almost identical on both compounds. *p < 0.01 treatment plus DME25 vs respective treatment without DME25 control; **p < 0.05 treatment plus DME25 *vs* respective treatment without DME25 control; ^#^p < 0.05 respective cells vs cells with control medium only. **c**, **d** Effects of DME25 on HGF and HGF receptor inhibitors, PHA665752 (**c**) and SU121274 (**d**), on HGF-induced invasion of A549 cells. *p < 0.01 treatment plus DME25 vs respective treatment without DME25 control; **p < 0.05 treatment plus DME25 *vs* respective treatment without DME25 control; # p < 0.05 respective cells vs cells with control medium only. The concentrations use for the test agents were: PHA665752—10 nM, SU11274—20 nM, HGF—50 ng/ml and DME25—1:1000 dilution

To evaluate the potential interplay between *YangZheng XiaoJi* and HGF/cMET, the effects of combining *YangZheng XiaoJi* and cMET kinase inhibitors, namely PHA665752 and SU11274, was tested. Using the EVOS method, it was found that *YangZheng XiaoJi* potentiated the effects of both inhibitors on cellular migration of both cells (Fig. 3A–D). Likewise, *YangZheng XiaoJi* also increased the inhibitory effect of both inhibitors on in vitro invasiveness of both cells (Fig. 3A–D). Overall, A549 seems to be more sensitive than SKMES1 in the response to HGF, the cMET inhibitors and to DME25.

**Fig. 4:**
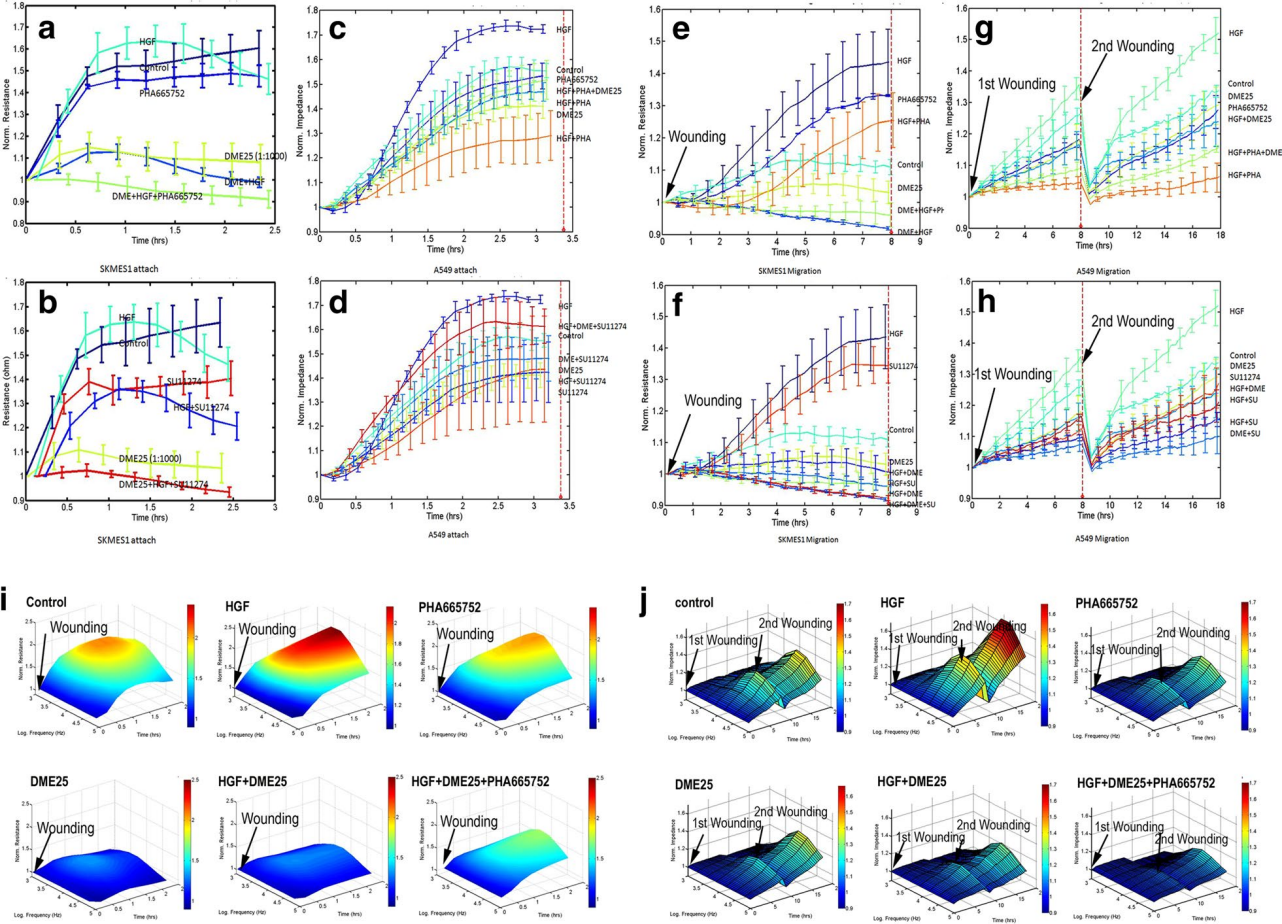
ECIS based tracking cell adhesion (**a**, **b**, **c**, **d**) and cell migration (**e**, **f**, **g**, **h**) for both SKMES1 (**a**, **b**, **e**, **f**) and A549 (**c**, **d**, **g**, **h**). **i**, **j** 3D modelling was used to display the response of cell migration over frequencies ranging from 1000 to 64,000 Hz. The experimental conditions for both adhesion and migration are similar to that in Fig. 2. **a**, **b** Adhesion of SKMES1 cells. HGF increased the migration, which was blocked by inclusion of DME25 and in particular by the combination of both DME25 and PHA665752 (**a**) and SU11274 (**b**). **c**, **d**: Adhesion of A549 cells. HGF had a marked effect on the adhesion of the cells, which was similarly reduced by DME25 and the cMET small inhibitors PHA665752 (**c**) and SU11274 (**d**). **e**, **f** Migration of SKMES1 cells following electric wounding. DME25, PHA665752 (**e**) and SU11274 (**f**) had a marked inhibitory effect on HGF induced cell migration. **g**, **h** Migration of A549 in a similar layout to (**e**, **f**), except the cell layer was wounded twice (at the beginning and after 8 h. **i** Adhesion of SKMES1 cells as demonstrated using a 3-dimensional model. The test was automatically carried out over a range of frequencies (1000 to 64,000 Hz). The inhibitory effect was seen across the range of frequencies applied in the study.** J** Migration of A549 cells shown in 3-D model. The A549 cells were wounded at the beginning and after 8 h. Wounding condition was 2wounded. *y-axis*: resistance (ohms); *x-axis*: frequencies in Hz; *z-axis*: time in hours. The concentrations of the test agents used here were the same as in Fig. 3

A similar observation was made when using the ECIS method in tracking cell adhesion (Fig. 4a–d) and cell migration (Fig. 4e–h) for both SKMES1 (a, b, e, f) and A549 (c, d, g, h). Figure 4 also demonstrates, in 3D imaging, the cell migration over a range of frequencies.

### YangZheng XiaoJi had an inhibitory effect on the phosphorylation of the HGF receptor, cMET

**Fig. 5:**
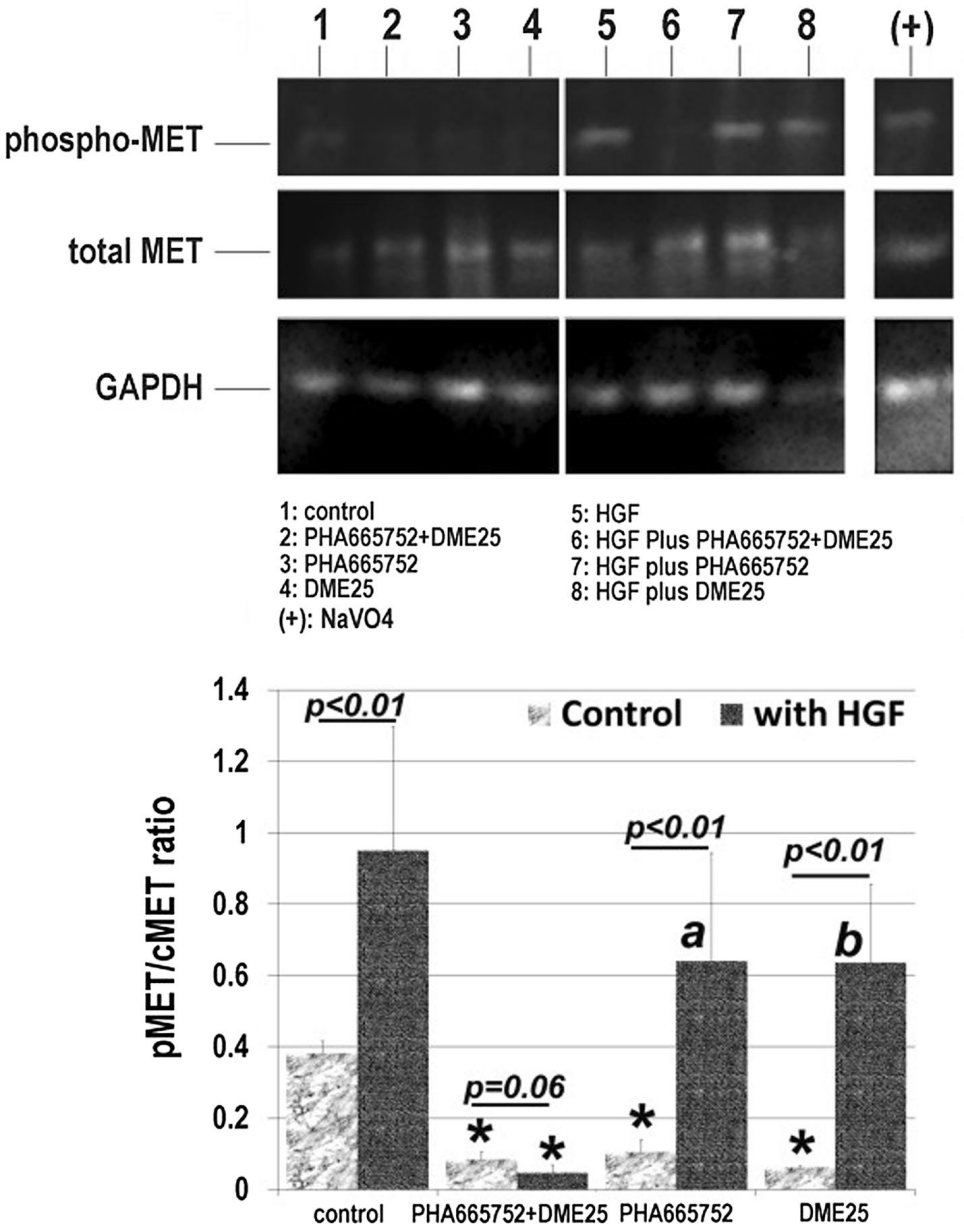
*YangZheng XiaoJi* had an inhibitory effect on the activation of the HGF receptor, cMET in lung cancer cell, A549. *Top* images from immunoblot assay. The phospho-MET (pMET), total MET (cMET) and the loading control GAPDH were probed with the respective antibodies. *Bottom* the pMET/cMET band density ratio for the immunoblots. The medicinal extract, DME25 was used at 1:1000 and cMET small inhibitor PHA665752 at 10 nM. Positive control for protein tyrosine phosphorylation was sodium orthovanadate (100 µM) with hydrogen peroxide (0.1 %). In quiescent cells, the limited activation of pMET was marked reduced by DME25, PHA665752 and the combination as indicated by *asterisk*. HGF caused marked action of the receptor (p < 0.01). The HGF induced activation was significantly inhibited by the DME25/PHA665752 combination (*asterisk*), however only marginally by DME25 alone (^a^p = 0.13 vs with HGF only) and PHA665752 (^b^p = 0.14, vs with HGF only)

The effect of *YangZheng XiaoJi* extract was further evaluated, directly or in combination with cMET small inhibitor PHA665752, on the action of the HGF receptor. In quiescent cells (4 h after serum hunger, only traceable amounts of pMET was detected in control cells (Fig. 5, top), which was significantly inhibited by DME25, PHA665752 and their combination (Fig. 5, top) (p < 0.01 vs control, Fig. 5, bottom). HGF resulted in a marked activation of the receptor (p < 0.01 vs without HGF control) (Fig. 5, bottom). Neither PHA665752 nor DME25 alone exhibited significant inhibition on the HGF activation of its receptor (p > 0.05). However, it was very interesting to note that the combination of PHA665752 and DME25 resulted in a significant inhibition in HGF-induced receptor phosphorylation (Fig. 5).

### Anti-tumour effects exerted by YangZheng XiaoJi in vivo

**Fig. 6:**
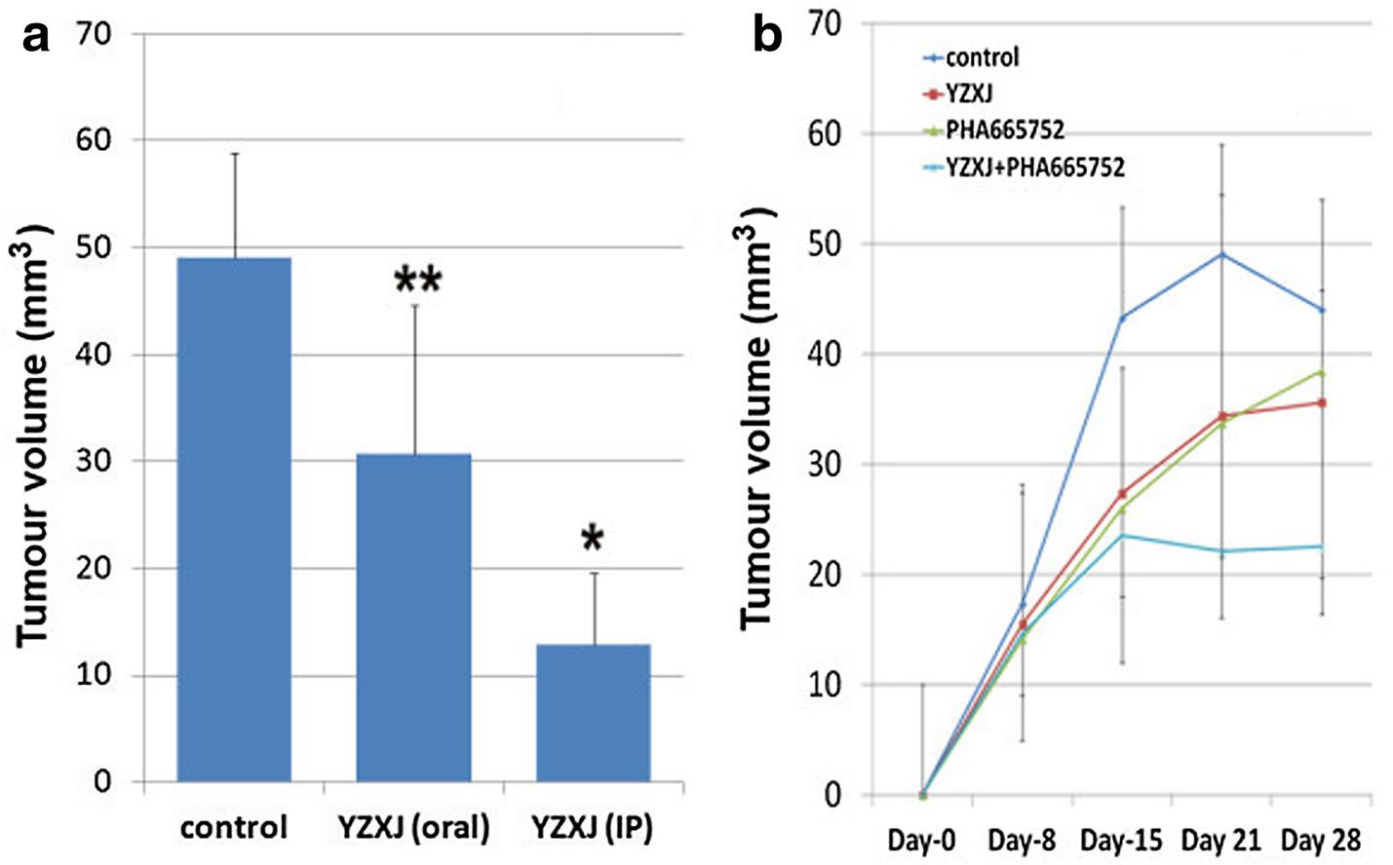
Effects of *YangZheng XiaoJi* and the cMET kinase inhibitor PHA665752 on the growth of A549 tumours. **a** Comparison between intraperitoneal and oral delivery of *YangZheng XiaoJi* on tumour growth, 3 weeks after treatments. IP route appears to be more effective than the oral route. *p < 0.01, **p = 0.05 vs control. **b** The effects of *YangZheng XiaoJi,* PHA665752 and their combination on the growth. All the treatments were via the oral route. The final concentration for *YangZheng XiaoJi* extract was 1 µl/g body weight and PHA665752 6.4 ng/g body weight. Tumour volume shown are mean ± SD in cubic milimeter

Using an in vivo A549 tumour model, it was shown that *YangZheng XiaoJi*, delivered daily, had a significant inhibitory effect on tumour growth (Fig. 6). The peritoneal route appeared to be more effective than the oral route (Fig. 6a). The cMET kinase inhibitor, PHA665752 also had an effect on slowing down the growth, in a similar manner as *YangZheng XiaoJi* (Fig. 6b). A combination of *YangZheng XiaoJi* and PHA665752 showed a more profound inhibitory effect on the growth of A549 tumours (Fig. 6b, the oral route of delivery of all the agents after 3 weeks treatment).

**Fig. 7:**
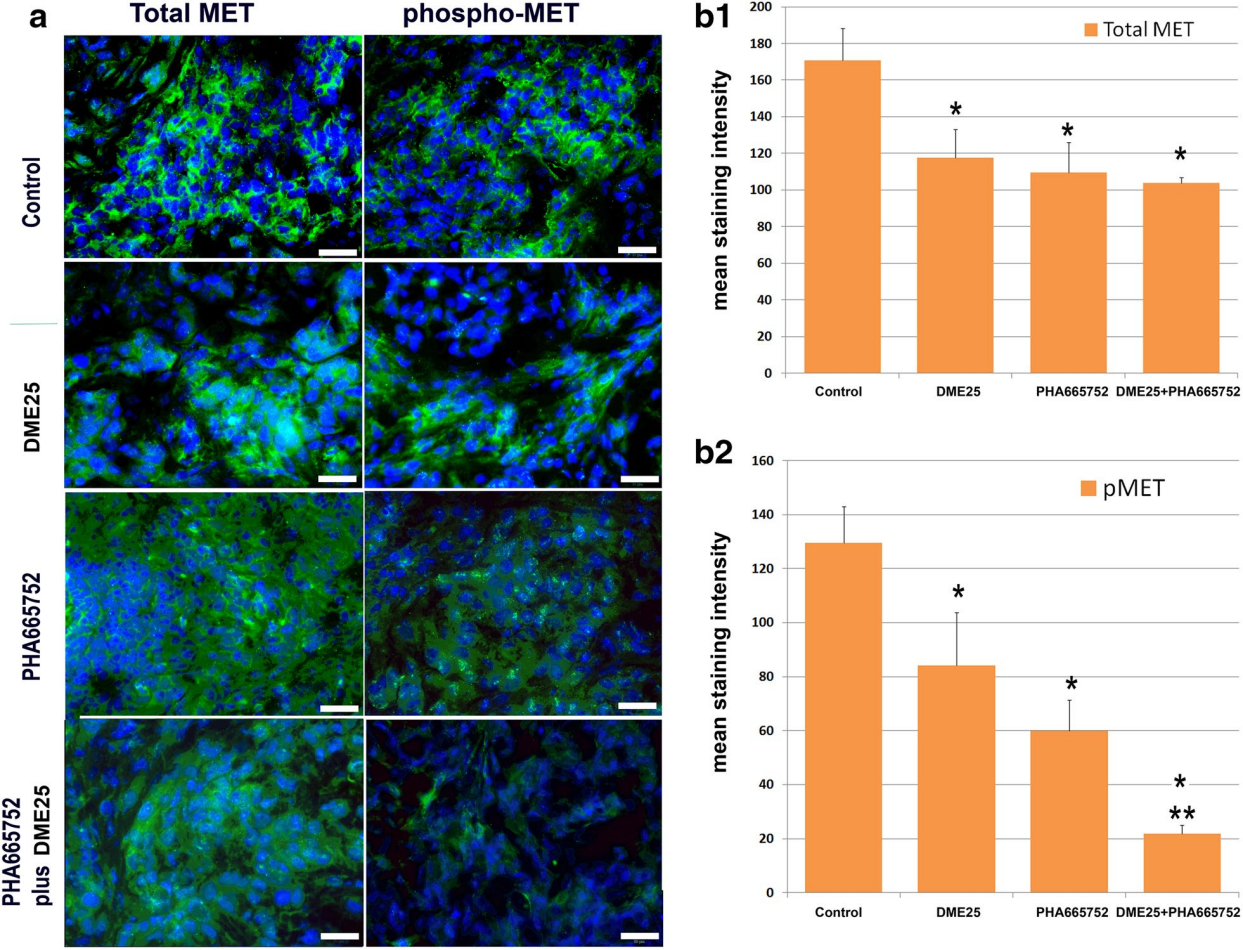
Immunofluorescence staining of total MET and phospho-MET (pMET 1384) in lung tumours from the in vivo model. **a** Immunofluorescence images of total MET (*left panel*) and phospho-MET (pMET 1384) (*right panel*) in lung tumours. Images were taken using an objective lens at ×40. *Scale bars* represent 50 µm. **b** quantified staining in the intercellular areas.** b1** total MET;** b2** phospho-MET (pMET). *p < 0.05 vs control; ** vs YZXJ alone and PHA665752 alone

Immunofluorescence staining for HGF receptor and the receptor activation further demonstrated the effect of the treatment on the HGF receptor. As shown in Fig. 7a, left and b1, the three treatments resulted in some degree of reduction of the staining of total MET. However, when the activated receptor was examined, results revealed both *YangZheng XiaoJi* and PHA665752, and in particular their combination, markedly reduced the intensity of the activation of MET (phospho-MET) (Fig. 7a, right and b2).

### YangZheng XiaoJi and the interplay between HGF and EGFR-TK complexes

**Fig. 8:**
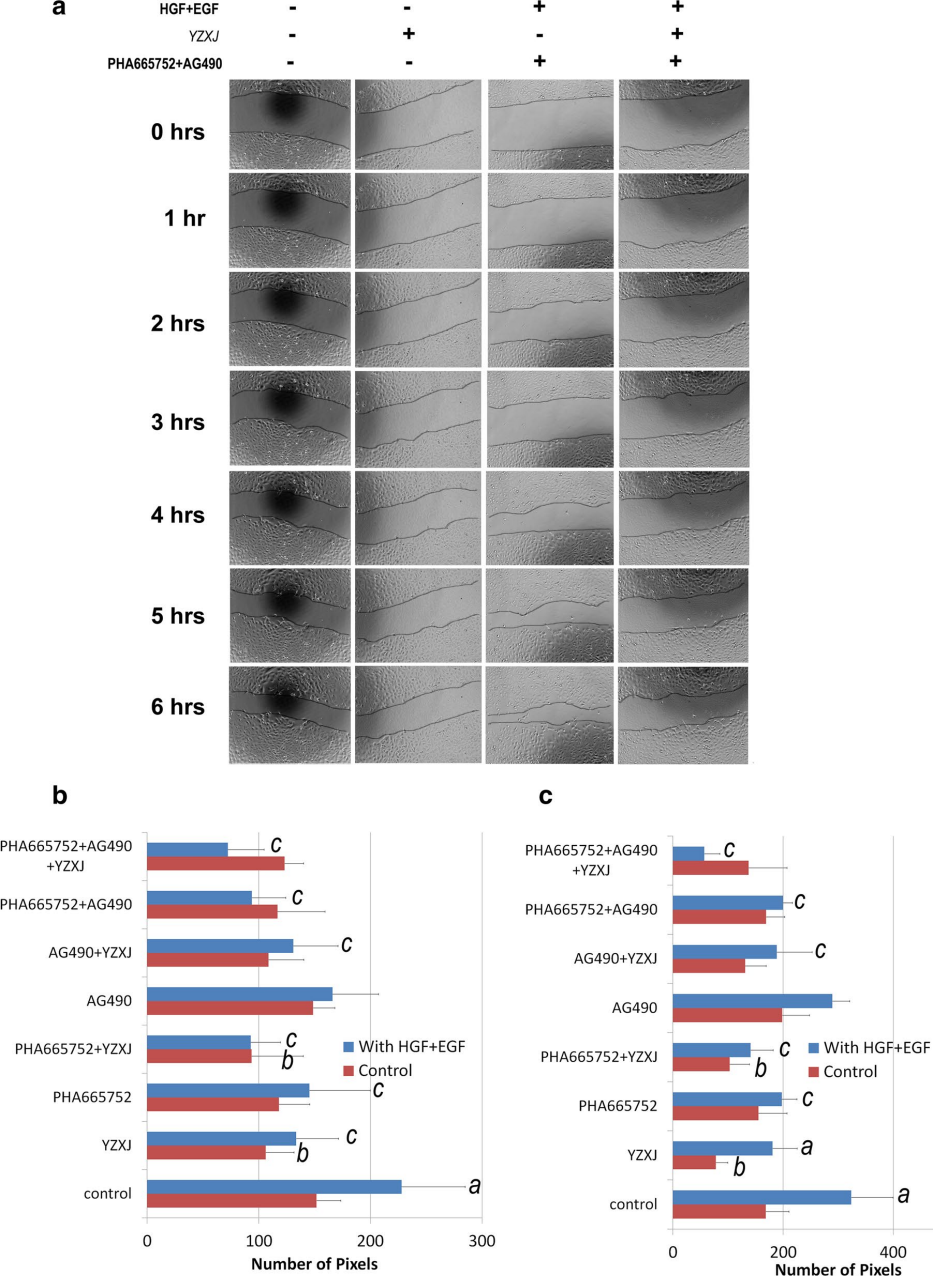
The interplay between HGF receptor, EGFR and YZXJ, as measured by cell migration. **a** A549 cellular migration tracked by EVOS. Cells were scratch-wounded and then added with *YZXJ* (1:1000), HGF (50 ng/ml)/EGF (10 ng/ml), the HGFR/EGFR inhibitors (PHA665752 10 nM and AG490 5 µM) alone or in combinations; **b** Migration distance for A549 cells; **c** Migration distance for SKMES1 cells. *a*: p < 0.05 vs no HGF/EGF; *b*: vs YZXJ alone; *c*: vs with HGF/EGF alone

In order to evaluate the possible interplay between *YangZheng XiaoJi* and the HGF/EGF axis, we further evaluated the effect of the medicine on HGF and HGF/EGF induced cell migration and cell growth. In cell migration assays, as shown in Fig. 8, the HGF/EGF combination markedly induced an increase in the migration of A549 cells (Fig. 8a, b) and particularly in that of SKMES1 cells (Fig. 8c). As demonstrated earlier, *YZXJ* was able to significantly inhibit the migration of both cells, both HGF/EGF treated and untreated cells. Inhibitor to the HGF receptor (PHA665752) at the concentrations used, showed a significant inhibitory effect on HGF/EGF induced cell migration. The effect of EGFR inhibitor (AG490) was somewhat less effective. The combination of PHA665752 with YZXJ and to a certain degree AG490 had a stronger effect. However, the strongest effects were seen when PHA665752, AG490 and YZXJ were combined, and this effect was particularly strong in HGF/EGF induced migration in SKMES1 cells.

## Discussion

In the present study, we have described for the first time that a traditional Chinese medicine formula, *YangZheng XiaoJi* has a profound direct effect on HGF induced aggressiveness of human lung cancer cells, both in vitro and in vivo. Furthermore, the medicine is also shown to inhibit the migratory effect induced by a combination of HGF and EGF, an axis of evil in clinical lung cancer in which they work synergistically in stimulating the progression of lung cancers.

HGF, and indeed its receptor cMET, are a pair of proteins that have been found to be frequently over-expressed in clinical lung cancers. In patients, raised levels of circulating HGF are frequently seen and, together with over-expression of HGF receptor, it is an evil pair of proteins causing the progression of lung cancer [8–21, 42]. HGF has some well established effects on cancer cells, including lung cancer cells. First, it is a powerful cellular migration and cellular invasiveness inducing factor, which earned it’s other name ‘scatter factor’; second, HGF is a potent angiogenic and lymphangiogenic factor; third, it acts as a mitogen for cells, although this is mainly seen in normal hepatocytes. There has been compelling evidence to show that they are very credible targets in cancer treatment. Indeed, the past few years has witnessed the development of therapeutic methods, namely small molecules and neutralising antibodies, to target HGF and its receptor, with some now being used in patients. HGF receptor inhibitors have been shown to reverse HGF induced EMT in lung cancer cells [19]. cMET over-expression has been shown to respond to cMET monoclonal (MAB) treatment as second line therapies by increasing overall survival and progression free survival [43]. In a recent study, PHA665752 has similarly been shown to block HGF induced effects on lung cancer cells [28]. Blocking HGF/cMET pathways by way of small kinase inhibitors, neutralising antibodies [44] and antagonist [45] have been shown to be viable new treatments for lung cancer.

The current study has shown that the Chinese medicine, *YangZheng XiaoJi,* has a strong inhibitory effect on HGF induced cellular migration of the lung cancer cells tested. The study has further demonstrated that *YangZheng XiaoJi* is able to work with the cMET inhibitor in a synergistic manner in extending the inhibitory effects on the migration of cancer cells. The effect appears to be replicated in the in vivo models, in which the combination of *YangZheng XiaoJi* and PHA665752, the cMET inhibitor, can markedly increase the inhibitory effects seen by individual agents alone. Thus, the study has presented a coherent observation that *YangZheng XiaoJi,* a traditional medicine currently being used in certain patients with cancer for example liver cancer, also has a therapeutic value in non-small cell lung cancer. In fact, in some small non-random studies, some benefits to the patients with lung cancer have already been reported although with little mechanistic studies.

The effects of *YangZheng XiaoJi* on the in vivo growth of lung cancer are more likely to be via multiple actions. The present study has reported a direct effect of the medicine on lung cancer cells. We have previously reported that the medicine also has a direct effect on angiogenesis in vitro and in vivo [10, 11]. Thus, it is plausible that the anti-tumour effects, in vivo, are via the direct action on cancer and on the microenvironment, namely angiogenesis. However, there have also been reports to show that patients who received the medicine have an improved immune function, for example favourable changes in NK cells and circulating cytokines. Thus, the anti-tumour and the therapeutic effect of the medicine are likely to be due to multiple factors.

The study has shown that application of *YangZheng XiaoJi* in tumour models has a significant effect on the levels of cMET, and most markedly activated cMET. In fact, both *YangZheng XiaoJi* and PHA665752 caused a reduction of cMET and phospho-cMET. The combination of both agents almost completely blocked the appearance of phospho-cMET in lung tumours. These observations suggest that blocking the HGF receptor activation is a key mechanism by which *YangZheng XiaoJi* has its biological influence on lung cancer cells. It has been previously reported that *YangZheng XiaoJi* also impacts on the focal adhesion kinase (FAK), the AKT and sonic hedgehog (SHH) pathways in cancer cells and vascular endothelial cells. The broad effect on multiple signalling pathways by *YangZheng XiaoJi* is not surprising, given that the medicine is a formula made of 16 herbal medicines. It is argued therefore that the medicinal formula of a mixture of ingredients would work on multiple targets. Future work to explore the nature of the ingredients would be highly desirable.

EGF receptor kinase inhibitors are now widely used in the treatment of lung cancer [7]. However, recent reports to show that HGF can trans-activate EGFR and make the anti-EGFR therapy less effective or indeed make lung cancer resistant to EGFR therapy in lung cancer [22–24] are very interesting indeed. Methods in targeting both have been suggested to be a viable option in enhancing the effect of anti-EGFR based therapies [46, 47]. Our study has shown that HGF and EGF had a profound effect on the migration of lung cancer cells. It is interesting to also note that AG490, an EGFR inhibitor has only marginal influence on this effect induced by HGF and EGF. We have shown that both *YangZheng XiaoJi* and PHA665752 are able to reduce the stimulation, and that a combination of the medicine, PHA665752 and AG490 almost completely abolished the effect of HGF/EGF. This indicates that combined use of *YangZheng XiaoJi* and HGF receptor inhibitor is an effective way to overcome resistance to anti-EGFR therapy in lung cancer and should be explored in clinical settings. Furthermore, it suggests that monitoring both HGF and EGF receptors are highly plausible in devising therapies for the patients.

## Conclusion

The present study has shown that *YangZheng XiaoJi* has some significant effects on the migration and invasion of lung cancer cells and in vivo tumour growth. The effect is particularly profound on the functions induced by HGF and the HGF/EGF combination and is attributable to the inhibition of HGF receptor activation. The medicine is particularly effective when used together with the HGF receptor inhibitors. It is concluded that *YangZheng XiaoJi* has a strong effect on HGF-induced cell adhesion and migration, which may be important when considering devising therapies in lung cancer and in the context of using the HGF/EGF receptor inhibitors in lung cancer.

## Additional file

**Additional file 1:** Chemical finger printing of 12 batches of *YangZheng XiaoJi*, demonstrating the consistency of the formula.

## Abbreviations

AG490: a small inhibitor to Epidermal Growth Factor Receptor
cMET: hepatocyte Growth Factor Receptor
DME25: a soluble extract from *YangZheng XiaoJi*
ECIS: electric cell-substrate impedance sensing
EGF: epidermal growth factor
EGFR: epidermal growth factor receptor
EVOS: a fl digital inverted fluorescence microscope
HGF: hepatocyte growth factor
NSCLS: non-small cell lung cancer
PHA665752: a small inhibitor to HGF receptor (cMET)
SCLS: small cell lung cancer
SU11274: a small inhibitor to HGF receptor (cMET)
TKI: tyrosine kinase inhibitor
*YZXJ*: *YangZheng XiaoJi*
