# Correction: YangZheng XiaoJi exerts anti-tumour growth effects by antagonising the effects of HGF and its receptor, cMET, in human lung cancer cells

**DOI:** 10.1186/s12967-023-04697-0

**Published:** 2024-01-04

**Authors:** Wen G. Jiang, Lin Ye, Fiona Ruge, Sioned Owen, Tracey Martin, Ping‑Hui Sun, Andrew J. Sanders, Jane Lane, Lucy Satherley, Hoi P. Weeks, Yong Gao, Cong Wei, Yiling Wu, Malcolm D. Mason

**Affiliations:** 1 Cardiff University‑Peking University Cancer Institute, Cardiff University School of Medicine, Henry Wellcome Building, Heath Park, Cardiff, CF14 4XN UK; 2 Yiling Medical Research Institute, No. 238 TianShan DaJie, Shijianzhuang, HeBei Province China

**Correction: J Transl Med (2015) 13:280** **https://doi.org/10.1186/s12967-015-0639-1**

Following publication of the original article [1], we have been notified that the conflict of interest declaration was incomplete. Co-authors Dr Y Gao, Dr C Wei and Dr Y Wu are employees of Yiling Medical Research Institute, a subsidiary research organisation of Yiling Pharmaceuticals and therefore are employees of Yiling Pharmaceuticals.

## References

[1] Jiang WG, Ye L, Ruge F, Owen S, Martin T, Sun PH, Sanders AJ, Lane J, Satherley L, Weeks HP, Gao Y, Wei C, Wu Y, Mason MD (2015). Epithelial cell adhesion molecule (EpCAM) regulates HGFR signaling to promote colon cancer progression and metastasis. J Transl Med.

