# Supplementary figures and images for: YangZheng XiaoJi exerts anti-tumour growth effects by antagonising the effects of HGF and its receptor, cMET, in human lung cancer cells
Source: J Transl Med. 2015 Aug 27;13:280. doi: 10.1186/s12967-015-0639-1 (PMC4551384; doi:10.1186/s12967-015-0639-1)

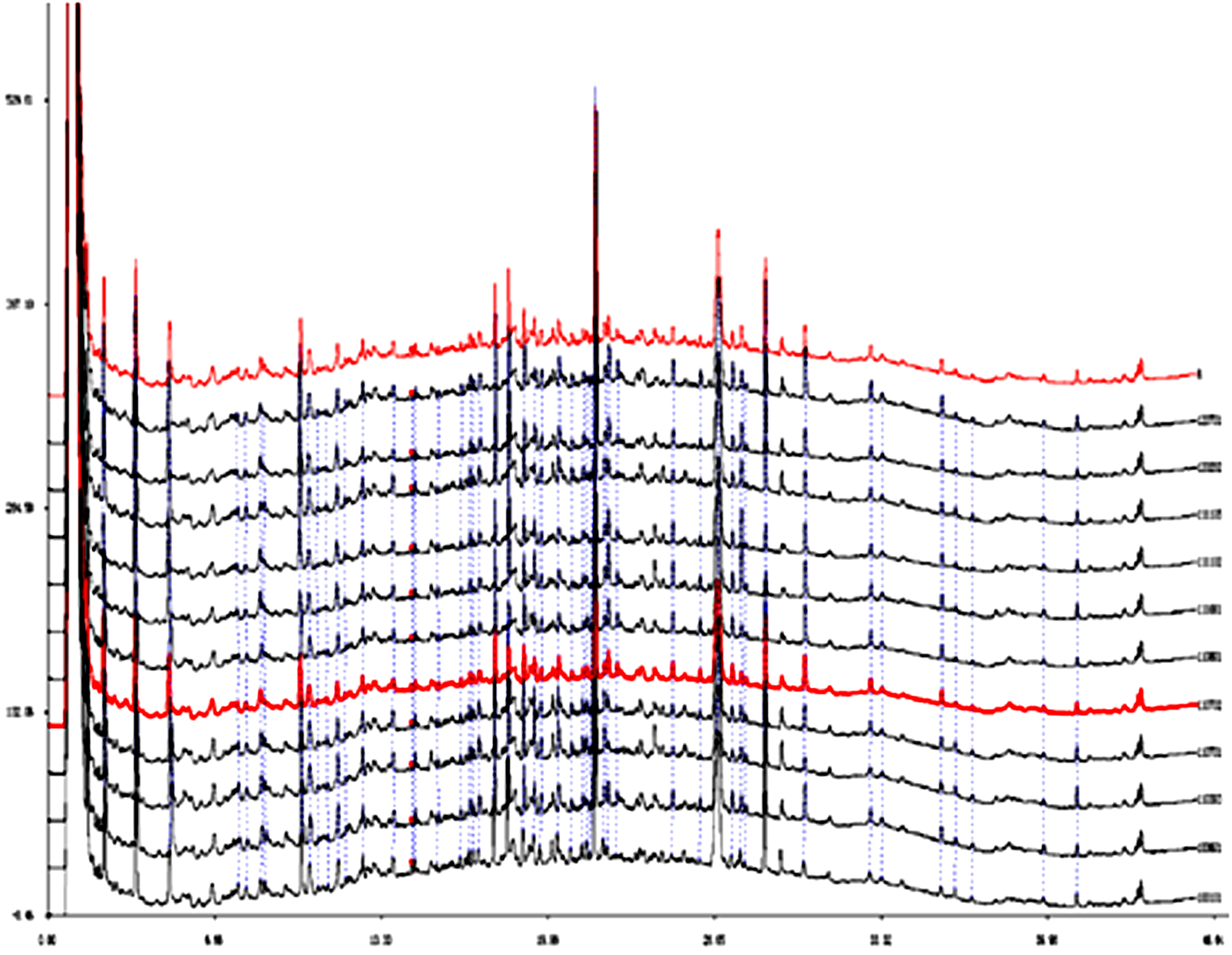

Supplement: Supplementary file 1 — Additional file 1: Chemical finger printing of 12 batches of YangZheng XiaoJi, demonstrating the consistency of the formula. [file 12967_2015_639_MOESM1_ESM.tiff]
